# Impact of Plant Part and Age of *Allium tuberosum* Rottler ex Sprengel on Antioxidant Properties

**DOI:** 10.3390/molecules31020332

**Published:** 2026-01-19

**Authors:** Agnieszka Żurawik, Aneta Wesołowska, Piotr Żurawik

**Affiliations:** 1 Department of Horticulture, West Pomeranian University of Technology in Szczecin, Słowackiego 17, 71-434 Szczecin, Poland; pzurawik@zut.edu.pl; 2 Department of Organic Chemistry and Physical Chemistry, West Pomeranian University of Technology in Szczecin, Piastów Ave. 42, 71-065 Szczecin, Poland; aneta.wesolowska@zut.edu.pl

**Keywords:** garlic chives, age, methanol extracts, GC–MS, antioxidant potential

## Abstract

*Allium tuberosum*, commonly known as garlic chives, is a promising species with significant antioxidant and anti-inflammatory properties, useful both fresh and dried as a spice. This study analyzed the chlorophyll, carotenoid, polyphenol content, and antioxidant activity in various parts of two- and three-year-old garlic chives, including green stems, inflorescences, and flowering shoots. The research found that flowering shoots had higher levels of chlorophylls and carotenoids, while inflorescences were rich in total polyphenols and exhibited the highest antioxidant activity. Essential oils extracted from different parts of the plant were analyzed using gas chromatography–mass spectrometry (GC–MS), revealing distinct chemical profiles. The oils contained unique compounds, with oxygenated monoterpenes predominant in green stems and stems with flower buds, and aliphatic hydrocarbons more prevalent in inflorescences. This study highlights the high antioxidant potential of *Allium tuberosum* and suggests further research due to its varied chemical composition across different plant parts.

## 1. Introduction

The health-promoting properties of plants, such as enhancement of the immune system [1], allow the plants to exert beneficial effects on human health [2]. A large number of people worldwide suffer from various diseases, including gastrointestinal disorders caused by numerous pathogenic bacteria such as *Bacillus cereus*, *Listeria* spp., *Escherichia coli*, and *Salmonella* spp., as well as other inflammatory conditions [3]. Antioxidants play a crucial role in maintaining human health [4]. Medicinal plants, as natural sources of bioactive compounds [5,6], also exhibit strong anticancer and antimutagenic activities, neuroprotective effects [7,8], reduce blood glucose and cholesterol levels [9], and influence gut microbiota [8]. Nour et al. [10] highlighted the health benefits of aromatic plants and the absence of any adverse effects. Natural antioxidants present in herbs help mitigate oxidative stress caused by gamma, UV, or X radiation; psycho-emotional stress; contaminated food; adverse environmental conditions; intense physical exertion; smoking; and alcohol consumption [11,12]. It has long been recognized that the role of herbs, as well as vegetables, in proper nutrition is substantial, primarily through their antioxidant activity and by providing a variety of nutritional and health-promoting compounds [13,14]. The number of conscious consumers seeking natural plant-based food additives, such as colorants or flavor enhancers, which are also sources of antioxidant compounds (interest in chlorophylls, carotenoids, and polyphenols is increasing) [9,10,12,15]. Aromatic plants represent an important source of chlorophyll, which exerts beneficial effects on human health as a natural, potent antioxidant [16]. Dried herbs, used as spices, stimulate appetite and positively influence human physiological functions [5], and they are an essential component in culinary preparation [17]. They are also a source of numerous biologically active compounds, including polyphenols [13,18,19], carotenoids—often referred to as “natural pesticides” [20]—and essential oils with antioxidant properties [13]. Aromatic plants and herbs exhibit significant potential in maintaining human health. Therefore, it is justified to expand their repertoire by exploring new plant species, particularly those recommended for organic cultivation, which whether fresh or dried (as spices) demonstrate high antioxidant and health-promoting value as well as appealing colors and aromas, making them suitable as culinary additives.

A highly promising species is *Allium tuberosum*, which belongs to the family *Amaryllidaceae* [21], which, in addition to compounds with antimicrobial and antibacterial properties contains numerous substances with antioxidant and anti-inflammatory activities, including polyphenols [3,22,23,24]. The antibacterial activity of garlic chives is attributed to the presence of sulfur-containing compounds [25], which also exhibit anticancer effects [26]. Furthermore, antifungal, antiparasitic, anticancer, hypolipidemic, and renoprotective activities of garlic chives have been reported [2], along with additional benefits such as stimulation of hair growth, regulation of hormonal balance, and aphrodisiac effects. This species is a popular vegetable in Asia and originates from China, where it is widely cultivated due to its significant medicinal properties [27]. The leaves and young inflorescences are the most commonly used parts of the plant [28]. It is also distinguished by its characteristic aroma [29]. *Allium tuberosum* is consumed as a fresh vegetable, dried, or cooked, and is added to various dishes, including kimchi and pickled cucumbers. The plant grows well even under unfavorable conditions [30] and does not require chemical protection against diseases and pests affecting bulb vegetables [31]. An important factor supporting its wider use is the possibility of utilizing all botanical parts (some of which are often considered by-products) in accordance with the zero-waste principle. 

There are no reports on the antioxidant properties of different botanical parts of *Allium tuberosum*, nor on whether these properties change with plant age. Therefore, this study was undertaken to determine the plant’s antioxidant potential by analyzing the contents of total polyphenols, carotenoids, chlorophyll a, chlorophyll b, and total chlorophyll, DPPH radical scavenging activity, and essential oils in various edible parts of garlic chives grown in the second and third growing season. It has been proposed that dried *Allium tuberosum* exhibits significant antioxidant activity; the level of activity varies among botanical parts and changes with plant age.

## 2. Results

The results of the study showed that all evaluated botanical parts of *Allium tuberosum* contain antioxidant compounds, consistent with the findings of Ašimović et al. [32], who reported that vegetables are a good source of phytochemicals. Statistical analysis indicated that the examined parts of garlic chives significantly differed in terms of total polyphenol content, total carotenoids, chlorophyll a, chlorophyll b, total chlorophyll, antioxidant activity, and essential oil content. Adamczewska-Sowińska and Turczuk [33] assessed the biological value of garlic chives in the second and third years of vegetation, reporting that this period represents the optimal time for *Allium tuberosum* to achieve its full biological value. Methanolic extracts obtained from different parts of garlic chives were examined and demonstrated to be rich sources of total polyphenols (Table 1). The highest total polyphenol content was found in the inflorescences, while the lowest was observed in the flowering stems and green stems of garlic chives, with differences of 6.34% and 11.8%, respectively. According to Caser et al. [34], this phenomenon is most likely associated with the production of specialized secondary metabolites by flowers, which play a key role in the reproductive process, as well as with the accumulation of defensive compounds that protect them from herbivores and pathogens. In plants grown in the third season, the highest polyphenol content was recorded in the inflorescences and the lowest in the stems, with a difference of 20.65%. The analyzed plant material contained on average 284.6 mg GAE/100 g of total polyphenols. Compared with the findings of Kopeć-Bieżanowska and Piątkowska [13], who reported in methanolic extracts the highest total polyphenol content in lemon balm (466.55 mg CAE/100 g) and the lowest in ginger (17.23 mg CAE/100 g), these results indicate a substantial richness of polyphenols in garlic chives.

In the study, the highest total carotenoid content was detected in the flowering stems, while the lowest was found in the inflorescences of garlic chives (Table 2). Similar relationships were reported by Lachowicz et al. [35] for *Allium ursinum*. With regard to total carotenoid content in flowers, Watkins [36] demonstrated that the content in white petals is markedly reduced. Higher total carotenoid levels were observed in the flowering stems of both the second and the third growing season, whereas lower levels in the stems in in the second season and inflorescences in the third one. The lowest levels in inflorescences were noticed in the second growing season. A significant relationship was also found between carotenoid content in the examined parts and plant age. Older, three-year-old plants contained 11.6% more carotenoids compared to two-year-old plants. Adamczewska-Sowińska and Turczuk [33] found 9.3% higher carotenoid content in the leaves of biennial garlic chives compared to triennial plants. These divergent results may suggest an influence of cultivation conditions [37]. A precise explanation of the relationship between carotenoid content and plant age indicates the need for further research.

Lachowicz et al. [35] reported an average value of 470.4 mg/kg DM of chlorophyll a and 741.4 mg/kg DM of chlorophyll b in the stems of wild garlic and concluded that these stems could be a significant dietary source of chlorophyll for humans. In contrast, the authors found considerably lower chlorophyll levels in the flowers of this species, averaging 299.1 mg/kg DM of chlorophyll a and 461.4 mg/kg DM of chlorophyll b. In the present study, comparing these plant parts in garlic chives, higher levels of chlorophyll a and b were also found in the stems compared to the inflorescences, with increases of 47.5% and 38.2%, respectively (Table 3). However, when we analyzed all examined parts of garlic chives, the highest amounts of chlorophyll a, b, and total chlorophyll were detected in the flowering stems, and the lowest in the inflorescences. Depending on the growing season, significantly higher contents of chlorophyll a, chlorophyll b, and total chlorophyll were observed in the flowering stems during both the second and third growing seasons, whereas the lowest contents were observed in the inflorescences of plants from both age groups. In two-year-old plants, this difference was substantial at 125.9 mg/kg DM, while in three-year-old plants it was 57.0 mg/kg DM. It is noteworthy that in garlic chives, higher amounts of chlorophyll a than b were detected in the examined parts, which is opposite to the pattern observed by [35] in wild garlic. According to Ašimović et al. [32], this may be related to external factors and environmental conditions, as chlorophyll a plays a central role in the photosynthetic process [8,38].

According to Adamczewska-Sowińska and Turczuk [33], the biological value of garlic chive leaves depends on plant age. They reported higher chlorophyll and carotenoid contentsw in the leaves of two-year-old garlic chives. In the present study, however, plant age did not affect the content of these parameters in stems, inflorescences, and flowering stems (Table 1 and Table 3).

Nergui et al. [39] reported the average antioxidant activity measured by the DPPH assay for fresh *Allium fistulosum* plants (77.8%) and *Allium cepa* (69.4%). In the present study, the average antioxidant activity of garlic chives was found to be high, at 69.8% (Table 4). The highest antioxidant activity was observed in the inflorescences of *Allium tuberosum*, and the lowest in the stems. The difference was substantial, amounting to 50.3%. These results suggest that the inflorescences of garlic chives possess a greater capacity to neutralize free radicals than flowering stems and green stems. A similar relationship was demonstrated by Tóth et al. [40] between the flowers and leaves of *Allium ursinum*.

Despite similar polyphenol contents in inflorescences and inflorescence stems, inflorescences exhibited a greater capacity to scavenge the DPPH radical. In contrast, green stems showed the lowest polyphenol content, which corresponded to the weakest DPPH radical scavenging activity.

From the fresh parts of garlic chives, light yellow oils with a characteristic garlic leaf-like aroma were obtained in yields of 0.02%, 0.08%, and 0.03% (*w*/*w*) for green stems, inflorescences, and stems with flower buds, respectively. A larger amount of essential oil (0.11% *w*/*w*) was obtained by Furletti et al. [41] from the fresh aerial parts of *A. tuberosum* cultivated in Brazil and by Lopes et al. [42] from the fresh leaves of *Allium tuberosum* cultivated in Brazil (0.3% *w*/*w*).

Differences in essential oil content between plants grown in Poland and Brazil may be due to several factors, including differences in the plant parts used, growing conditions, differences in extraction methods, and plant maturity at harvest. Furthermore, the higher oil yields obtained from fresh aerial parts and leaves of plants grown in Brazil may also suggest that the growing conditions in Brazil are more favorable for essential oil production in garlic chives.

The chemical composition of the essential oils (EOs), the percentage content and retention indices of the constituents, and the main classes and chemical groups of the identified compounds are summarized in Table 5.

A total of 102 compounds were identified in the garlic chives’ stem oil, representing 99.10% of the total oil. The EO consisted mainly of allyl methyl trisulphide (15.26%), linalool (8.00%), diallyl disulphide (7.33%), and dimethyl trisulphide (6.50%).

Concerning the inflorescences’ oil, 91 compounds representing 98.63% of the total EO were identified. The major components were allyl methyl trisulphide (11.58%), 1-pentacosene (7.51%), diallyl disulphide (6.86%), 1-tricosene (5.82%), diallyl trisulphide (5.71%) and 1-heptacosene (5.22%).

One hundred and two constituents, which represented 99.29% of the total oil, were identified in the oil from stems with flower buds. Allyl methyl trisulphide (17.28%) was the major component, followed by dimethyl trisulphide (12.84%), diallyl disulphide (10.00%), allyl methyl disulphide (9.78%), and linalool (8.25%).

Considering the identified compounds, only 53 of them were common for all the oils. The obtained results showed that the chemical composition of *A. tuberosum* clearly depends on the part of the plant analyzed. Decane, carvone, 2-phenyl-2-butenal, tridecane, methyl cinnamate, β-cubebene, β-gurjunene, *cis*-3-hexenyl benzoate, τ-muurolol, α-eudesmol, pentadecanal, pentadecanoic acid, 1-nonadecene, palmitic acid, methyl linoleate, methyl stearate, linoleic acid, 6-methylhexacosane, and squalene were detected only in the stem oil, while *trans*-hex-3-en-1-ol, *trans*-2-octen-1-ol, nonyl acetate, bicycloelemene, 2-tridecanone, α-muurolol, 5-ethyl-5-methylpentadecane, benzyl benzoate, hexadecanal, 13-epi-manool, 1-docosene, eicosanal, *trans*-5-eicosene, 1-tetracosene, 5,5-dimethylheneicosane, docosanal, 11-tricosene, 2-methylpentacosane, 3-methylpentacosane, methyl lignocerate, 5,17-dimethylheptacosane, 2-methyloctacosane, and 1-tricontene were present only in the oil extracted from inflorescences. Compounds such as 2-ethylpyridine, *cis*-hex-3-en-1-ol, heptanal, 1-octen-3-ol, 3-octanol, *p*-cymene, 2,4-octadienal, β-elemene, β-bourbonene, *trans*-nerolidol, 2-methylheptadecane, *trans*-α-atlantone, and 3-methylheptadecane we found only in the oil obtained from stems with flower buds.

The percentage composition of different groups of compounds in *Allium tuberosum* essential oils (EOs) has been thoroughly examined (refer to Table 5). Oxygenated monoterpenes were identified as the primary constituents in the oils from green stems (15.96%) and stems with flower buds (12.13%), whereas aliphatic hydrocarbons (26.41%) were predominant in the oil from inflorescences. The content of monoterpene hydrocarbons in all the oils did not exceed 0.5%. Additionally, the amount of sesquiterpene hydrocarbons was found to be low in the oils from green stems (2.93%), inflorescences (4.13%), and stems with flower buds (1.61%). Similarly, the levels of oxygenated sesquiterpenes were low, recorded at 0.68%, 4.06%, and 3.12% for green stems, inflorescences, and stems with flower buds, respectively. Furthermore, the content of phenols (2-methoxy-4-vinylphenol and eugenol) in all oil samples was very low, ranging from 0.51% to 0.91%. Estragole, a representative of phenylpropanoids, was detected only in the EOs of green stems (1.96%) and stems with flower buds (0.87%). Conversely, the volatile oil from green stems exhibited a higher content of fatty acids (5.83%) and fatty acid esters (5.27%).

The variations in the composition of essential oils from different parts of *Allium tuberosum* can be attributed to the distinct biochemical pathways and functions associated with each plant part. The stems, inflorescences, and stems with flower buds each fulfill unique roles within the plant, leading to a divergence in the synthesis of specific compounds. For instance, the predominance of oxygenated monoterpenes in the oils from green stems and stems with flower buds suggests a role in deterring herbivores or attracting pollinators, while the higher concentration of aliphatic hydrocarbons in inflorescences may be related to structural or protective functions. The presence of specific compounds only in certain parts, such as estragole in green stems, indicates localized biosynthetic activity tailored to the plant’s developmental or environmental needs. Additionally, the observed differences in fatty acids and esters content reflect the metabolic specialization of each plant part, possibly linked to energy storage or membrane structure [45,46].

The volatile compounds found in the parts of *Allim tuberosum* may also be categorized into sulphides, disulphides, and tetrasulphides.

As shown in Table 6, sulfur-containing compounds account for 53.12, 45.28, and 71.43% of the total volatiles of green stems, inflorescences, and stems with flower buds, respectively.

The highest content of dimethyl trisulphide (12.84%) was found in stalks with flower buds, while the lowest content of this constituent was noted in the inflorescences (4.72%). Moreover, the stalks with flower buds had the highest content of allyl methyl disulphide (9.78%), methyl propyl disulphide (1.23%), methyl 1-propenyl disulphide (3.79%), diallyl disulphide (10.00%) and dimethyl tetrasulphide (3.05%). According to the results, the highest content of diallyl trisulphide (5.71%), allyl methyl tetrasulphide (3.17%) and diallyl tetrasulphide (0.93%) were found in the inflorescences. When comparing the methional content, it was shown that its highest value was in the inflorescences (0.89%), while its lowest value was noted for stalks with flower buds (0.31%). Although, the highest value of methyl-1-(methylthio)ethyl-disulphide was noted for the essential oil isolated from the green stems, while the lowest content of this constituent was noted in the inflorescences. Trace amounts of methyl *trans*-1-propenyl trisulphide (0.08%) and di-1-propenyl trisulphide (0.06%) were found in green stem oil, while diallyl sulphide (0.05%) was present only in the oil obtained from stalks with flower buds. Interestingly, di-2-propenyl tetrasulphide (0.19%) was noted only in the oil of inflorescences.

According to literature data, the characteristic odor of *A. tuberosum* is due to the presence of sulfur-containing flavor components.

Mnayer et al. [43] identified dimethyl disulphide (19.58%), allyl methyl disulphide (14.37%), dimethyl trisulphide (14.34%), allyl methyl trisulphide (7.24%), methyl 1-propenyl disulphide (6.07%), and diallyl disulphide (5.14%) in the essential oil obtained from bulbs of garlic chives from France. The main components of the essential oil from *Allium tuberosum* cultivated in Brazil were allyl methyl disulphide (25.9%), diallyl disulphide (22.5%), and dimethyl disulphide (5.3%) [42], while Pino et al. [47] found methyl propyl trisulphide (9.9%), dipropyl trisulphide (6.0%), dimethyl trisulphide (6.0%), and methyl propyl disulphide (5.5%) in volatiles from Chinese chives cultivated in Havana.

The essential oils composition detailed in this study shows some differences from previously published reports on garlic chives [42,43,47]. Dimethyl disulphide, which dominated in the essential oil from garlic chives cultivated in France [43] and in Brazil [42] was not detected in our oils. The content of components such as dimethyl trisulphide, allyl methyl disulphide, dimethyl trisulphide, allyl methyl disulphide, methyl propyl trisulphide, dipropyl trisulphide, and diallyl disulphide found in the oils from current study was lower as compared to cited literature. It can be concluded that the relative proportions of sulfur-containing compounds in garlic chives essential oil vary significantly depending on the collection places and botanical organs used for extraction.

Aliphatic aldehydes, ketones, and aliphatic hydrocarbons were found in *Allium tuberosum* leaves and seeds by Lopes et al. [42] and Hu et al. [48]. Moreover, the presence of other volatiles like furfuryl and furan derivatives, phenols, diterpenes, and terpenoids in different genotypes of *Allium tuberosum* have been confirmed by Hanif et al. [49]. However, linalool, which we found in significant amounts (3.16–8.25%) in our oils, was present in lesser amounts (1.75%) in *Allium tuberosum* from France [43].

This study provides valuable information on the antioxidant properties of dried *Allium tuberosum*, which can be used as a spice or ingredient in food products beneficial to human health. The essential oils derived from garlic chives, particularly from different parts of the plant, exhibit a diverse range of chemical compounds that contribute to their biological activities. Notably, allyl methyl trisulphide, diallyl disulphide, and dimethyl trisulphide are prominent constituents known for their antimicrobial and antioxidant properties. These sulfur-containing compounds have been widely studied for their ability to inhibit the growth of various bacteria and fungi, making them valuable in food preservation and as potential therapeutic agents. Linalool, another significant component, is recognized for its calming and anti-inflammatory effects, often utilized in aromatherapy and skincare products [45,50,51]. Furthermore, the distinct aroma of garlic chives, attributed to its sulfur compounds, can enhance the flavor profile of culinary dishes, making it a valuable ingredient in the food industry. The diverse chemical composition also suggests potential applications in the agricultural sector as a natural pest deterrent, leveraging the plant’s ability to repel herbivores. Moreover, the exploration of garlic chives essential oils in pharmaceuticals could yield new formulations for anti-inflammatory or antimicrobial treatments.

## 3. Materials and Methods

### 3.1. Field Experiment

The field experiment was conducted from 2022 to 2024 at the West Pomeranian University of Technology in Szczecin (14°31′ E, 53°26′ N), within a vegetative hall. The experimental factors were the botanical part of the plant and the plant age.

The garlic chive seeds used in the study were obtained from the commercial seed company PlantiCo, Zielonki Poland. Garlic chives were cultivated from unpotted seedlings produced in an unheated greenhouse. Untreated seeds were sown on 14 March 2022 (Season 1) into seed trays filled with a substrate based on high peat with a pH of 5.4–6.0 containing the macronutrients NPK (14–16–18) + Mg (5) at a rate of 0.6 kg/m^3^ and micronutrients at 0.2 kg/m^3^. The seedlings were subsequently transplanted on 29 April to their permanent location in perforated plastic trays measuring 60 × 40 × 24 cm, planted in nests with five seedlings per nest, at a spacing of 30 × 20 cm. Each tray contained four clumps of garlic chives. The sides of the trays were covered with black foil. The trays were filled with a light mineral soil with a pH of 6.7, in a volume of 45 L. The experiment was established in a randomized sub-block design with three replications, each consisting of five trays. In total, the study was conducted on 60 plant clumps. Nutrient deficiencies were supplemented to the level recommended for *Allium cepa* [52] using the multi-component fertilizer Azofoska (N 13.6, P_2_O_5_ 6.4, K_2_O 19.1, MgO 4.5, B 0.045, Cu 0.180, Fe 0.17, Mn 0.27, Mo 0.040, Zn 0.045). In the first year of cultivation, the fertilizer was applied to each tray twice: the first application was three weeks before the planned seedling transplanting at a dose of 25 g, and the second application was four weeks after transplanting at a dose of 20 g. In subsequent years, the plants were fertilized with the same fertilizer once, four weeks after the onset of the growing season, at a dose of 20 g. Plant harvests for laboratory analyses began in the second year of cultivation, i.e., in 2023 (season 2). Every 10 days, flowering stems with buds and fully developed flowers in the inflorescences were randomly harvested separately. No leaf harvests were conducted during the growing season. In the first year of the study (season 2), the flowering stems were harvested four times: 5 September, 15 September, 20 September, and 1 October. In the second year (season 3), they were harvested six times: 15 August, 25 August, 5 September, 16 September, 25 September, and 7 October. *Allium tuberosum* is a perennial plant cultivated over several growing seasons. The variation in the number of harvests resulted from differences in the growing season. Older plants initiated flowering earlier and completed flowering later than younger plants. The number of harvests did not affect the laboratory evaluation, as the entire yield of inflorescences from both growing seasons of garlic chives was subjected to analysis. Properly developed inflorescence stems were harvested when approximately 75% of the flowers had developed, at a height of 5 cm above the soil surface. The harvested material was divided into flowering stems, green stems, and inflorescences.

During plant growth and development, basic maintenance practices were performed, including weeding and irrigation. In both years of the study, there was no need to apply chemical protection against diseases and pests affecting bulb vegetables. In each study year, after the end of the growing season, leaves were cut at a height of approximately 5–7 cm above the soil surface in November. The analysis of weather conditions in 2023–2024 was based on data obtained from the Institute of Meteorology and Water Management for the Hydrological-Meteorological Station IMGW in Szczecin-Dąbie (Table 7).

Under the climatic conditions of Szczecin, garlic chives begin producing flowering stems in July and bloom until the end of September or early October. The most intensive flowering period occurs from late July to early September. In both study years, weather conditions were favorable for the growth of this species. During flowering, air temperature and sunshine levels were at or above the long-term average. The plants flowered very abundantly. In contrast, monthly precipitation totals, compared to long-term averages, were higher in July in both study years, whereas in August and September, lower precipitation was recorded in the second year of the study.

### 3.2. Laboratory Analyses

Plant material analyses were carried out in three replications in the laboratories of the Department of Horticulture and the Department of Organic and Physical Chemistry at the West Pomeranian University of Technology in Szczecin. For each batch of raw material, ten parallel measurements were performed.

**Figure 1:**
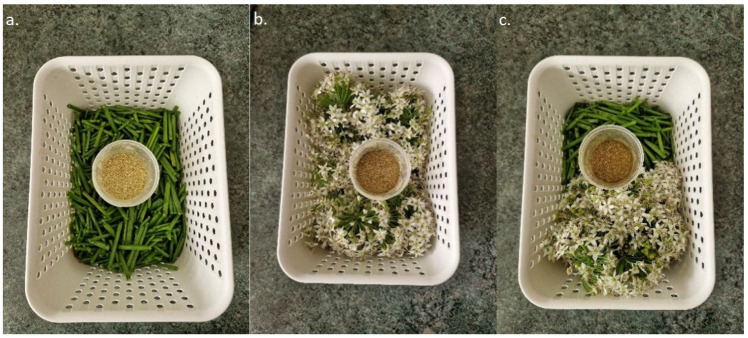
Fresh and dried plant material (**a**) green stems, (**b**) inflorescences, (**c**) flowering stems (photo by A. Żurawik).

The contents of total polyphenols, chlorophyll a, chlorophyll b, total chlorophyll, total carotenoids, and DPPH radical scavenging activity in green stems, inflorescences, and flowering stems were determined in dried material (Figure 1). Flowering stems (35 g), green stems (30 g), and inflorescences (40 g) were dried separately using an SLN 115Eco laboratory dryer (POL-ECO, Wodzisław Śląski, Poland) at 40 °C. After drying, the material was ground using a WŻ-1 laboratory mill (Instytut Sadkiewicza, Bydgoszcz, Poland). This procedure was repeated after each harvest. Subsequently, the dried material from different plant parts was combined into composite samples from individual harvests and subjected to chemical analyses. The same procedure was applied in the second year of the study. In both years of the study, dried material with a particle size of 0.8 mm was obtained. The moisture content of the material was as follows: green stems (9.25% and 9.68%), inflorescences (11.27% and 11.60%), and flowering stems (13.35% and 12.82%).

Essential oils in green stems, inflorescences, and stems with floral buds were analyzed in fresh plant material collected on 25 September 2024.

#### 3.2.1. Determination of Chlorophylls and Carotenoids

Plant material (0.5 g) was extracted with 80% acetone. The samples were ground in a mortar with a small amount of acetone and then transferred to a 50 mL volumetric flask. The residue was rinsed with acetone until the extracted material was completely decolorized. The extract was analyzed using a Helios Gamma spectrophotometer (Thermo Spectronic, Rochester, NY, USA) at wavelengths of 441, 646, 652, and 663 nm, with three replications.

The pigment content in the analyzed material was calculated using the following formulas:

$$\frac{mg\;chlorophyll\;a}{kg}=\left(12.21\times E_{663}-2.81\times E_{646}\right)\times \left(\frac{V}{m}\right)$$

$$\frac{mg\;chlorophyll\;b}{kg}=\left(20.13\times E_{646}-2.81\times E_{663}\right)\times \left(\frac{V}{m}\right)$$

$$\frac{mg\;total\;chlorophyll}{kg}=\left(27.8\times E_{652}\right)\times \left(\frac{V}{m}\right)$$

$$\begin{array}{c} \frac{mg\;total\;carotenoids}{kg}=[\left(1000\times E_{441}\right)-3.27\times \left(12.21\times E_{663}-2.81\times E_{646}\right)-\\ 104\times \left(20.13\times E_{646}-5.03\times E_{663}\right)]\times \left(\frac{V}{m\times 229}\right) \end{array}$$

where *E* is the extinction at the specified wavelength, *V* is the volume of the volumetric, and *m* is the mass of the sample.

#### 3.2.2. Determination of Total Polyphenols

The total polyphenol content was determined spectrophotometrically using the Folin–Ciocalteu reagent, with gallic acid as the standard [53]. Plant material (1.0 g) was extracted for 30 min with 70% methanol. The cooled extract was then quantitatively transferred to a 100 mL volumetric flask using methanol. The flask was stoppered and mixed thoroughly. To obtain the final extract, the sample was filtered. Using a pipette, 5 mL of the extract was transferred to a 100 mL volumetric flask, and the following were added sequentially: 75 mL of distilled water, 5 mL of Folin–Ciocalteu reagent, and 10 mL of saturated Na_2_CO_3_ solution. The flask was filled up to 100 mL with distilled water and mixed. The sample was kept at 21 °C for 60 min in the dark. The absorbance was measured using a Helios Gamma spectrophotometer (Thermo Spectronic) at a wavelength of 760 nm.

The total polyphenol content was calculated using the formula:

$$P=\frac{\left(y\times 100\right)}{A}$$

where *A* is the weight of the plant material divided by 20 and *y* is the value obtained from the standard curve.

Polyphenol concentration was expressed as milligrams of gallic acid equivalents (GAE) per 100 g of dry matter (DM). 

#### 3.2.3. Determination of Antioxidant Activity Using the DPPH Method

The antioxidant activity of the plant material was determined using the DPPH radical scavenging method [54]. Prior to the analysis, a reagent containing the radical solution was prepared. A sample of 0.012 g DPPH (2,2-diphenyl-1-picrylhydrazyl) was weighed and quantitatively transferred to a 100 mL volumetric flask, which was then filled to the mark with methanol. The test sample was prepared in a test tube by sequentially combining: 1 mL of a fivefold diluted methanolic plant extract, 3 mL of methanol, and 1 mL of the DPPH solution. The mixture was shaken, and after 10 min the absorbance was measured at 517 nm using a spectrophotometer. The percentage of DPPH inhibition was calculated using the formula [55]:

$$\%DPPH\;inhibition\;=\;\left[\frac{\left(Ar-At\right)}{Ar}\right]\;\times \;100$$

where *Ar* is the absorbance of the control and *At* is the absorbance of the test sample.

### 3.3. Determination of Essential Oil Content

Separate 100 g samples of stems, inflorescences, and stems with floral buds were weighed and placed in a 1000 mL round-bottom flask with 400 mL of distilled water. The samples were subjected to hydrodistillation using a Clevenger-type apparatus (Labo24.pl, Gliwice, Poland) for 4 h, according to the method recommended in the European Pha(rmacopoeia [56], with some modifications. After this time, the condensate collected into the calibrated tube of the apparatus was extracted once with 25 mL of dichloromethane to completely recover the oil (the content of oil in the garlic chives was so small that it did not form a separate layer on the water surface). Anhydrous sodium sulfate was added to the dichloromethane to remove moisture. Next, the dichloromethane was removed by rotary evaporation at 40 °C to give light yellow oils with the characteristic chive smell. The obtained oils were stored in tightly closed vials at 4 °C until analysis. The essential oil content was determined and expressed as weight of oil per fresh material weight (%*w*/*w*).

#### GC–MS Analysis

GC–MS analysis of the isolated oils was realized on an HP 6890 gas chromatograph equipped with an HP-5 MS fused silica column (30 m × 0.25 mm × 0.25 μm film thickness) and directly coupled with an HP 5793 mass selective detector (Agilent Technologies, Foster City, CA, USA). Helium was used as a carrier gas at a flow rate of 1.0 mL/min. The volume of sample injected was 2 μL (20–30 mg of oil dissolved in 1.5 mL of dichloromethane) and split injection was used (split ratio 5:1). The injector and the transfer line were kept at 250 °C and 280 °C, respectively. The ion source temperature was 230 °C.

The oven temperature was raised from 50 °C to 280 °C at a rate of 3 °C/min. Mass spectra were taken at 70 eV with a mass scan range of 50–550 amu.

Solvent delay was 4 min. The total running time for a single sample was 76.76 min.

Most of the constituents were identified by comparison of their mass spectra with the spectrometer databases (Wiley NBS75K.L and NIST 2002) and by comparison of their calculated retention indices with those reported in the NIST Chemistry WebBook (https://webbook.nist.gov/chemistry/, accessed on 15 November 2025) and literature [43,44]. The retention indices were determined in relation to a homologous series of n-alkanes (C_7_–C_30_, from Supelco, Bellefonte, PA, USA) under the same operating conditions. Each GC–MS analysis was repeated three times.

The average values of the relative percentages of essential oil constituents were determined from the total peak area (TIC) using MSD Enhanced ChemStation G1701AA ChemStation A.03.00. software.

### 3.4. Statistical Analysis

The results of the chemical analyses, including total polyphenols, chlorophyll a, chlorophyll b, total chlorophyll, total carotenoids, DPPH radical scavenging activity, and essential oils in different botanical parts of two- and three-year-old *Allium tuberosum* (season 2 and 3), were statistically processed using Statistica Professional 13.3 (TIBCO StatSoft, Palo Alto, CA, USA) and verified by two-way analysis of variance (ANOVA) for each plant part and study year. Mean values were compared using Tukey’s test at a significance level of *p* ≤ 0.05.

## 4. Conclusions

*Allium tuberosum* is a valuable plant in terms of total polyphenol content, total carotenoids, chlorophyll a, chlorophyll b, total chlorophyll, antioxidant activity, and essential oil content across all examined botanical parts. The inflorescences of garlic chives were particularly rich in total polyphenols and exhibited high antioxidant activity. Flowering stems, in turn, represent the best source of total carotenoids and chlorophyll a, b, and total chlorophyll. Three-year-old plants contained 11.6% more carotenoids compared to two-year-old plants. A total of 154 essential oil components were identified in the different botanical parts of garlic chives. In the volatile profile of garlic chives cultivated in northwestern Poland, sulfur-containing compounds predominated. The highest content of these compounds was recorded in the essential oil isolated from stems with floral buds (71.43%), and the lowest in the essential oil of the inflorescences (45.28%). Both two- and three-year-old garlic chives were rich sources of antioxidants and may be recommended for use in the food industry. A more comprehensive phytochemical characterization could strengthen the potential application of garlic chives as a functional food. Furthermore, to enable the commercialization of *Allium tuberosum* in food products, it is necessary to optimize harvesting, drying, and storage technologies to preserve its maximum antioxidant properties.

## Figures and Tables

**Table 1:** The content of polyphenols (mg GAE/100 g) in garlic chives, depending on the tested part and age of the plant.

Tested Part of the Plant	Season 2	Season 3	Mean
green stem	272.11 ± 1.81 ^cde^*	247.06 ± 1.89 ^f^	267.1 ^B^
inflorescence	294.41 ± 5.44 ^bcd^	311.35 ± 2.03 ^a^	302.9 ^A^
inflorescence stem	282.54 ± 1.64 ^cde^	284.91 ± 1.15 ^cde^	283.7 ^AB^
Mean	283.0 ^A^	286.1 ^A^	-

* The results were expressed as the mean ± standard deviation (SD). Different lowercase letters indicate statistically significant differences (*p* ≤ 0.05) between the tested plant parts and plant ages, whereas different uppercase letters denote significant differences (*p* ≤ 0.05) for the main factor.

**Table 2:** The content of carotenoids (mg/kg DM) in depending on the tested part and age of garlic chives.

Tested Part of the Plant	Season 2	Season 3	Mean
green stem	169.90 ± 0.23 ^cde^*	179.06 ± 0.83 ^abcde^	174.5 ^B^
inflorescence	95.88 ± 1.27 ^f^	157.76 ± 3.74 ^cde^	126.8 ^C^
inflorescence stem	221.78 ± 1.68 ^abc^	214.74 ± 0.97 ^abc^	218.3 ^A^
mean	162.5 ^B^	183.9 ^A^	-

* The results were expressed as the mean ± standard deviation (SD). Different lowercase letters indicate statistically significant differences (*p* ≤ 0.05) between the tested plant parts and plant ages, whereas different uppercase letters denote significant differences (*p* ≤ 0.05) for the main factor.

**Table 3:** The content of assimilation pigments (mg/kg DM) in depending on the tested part and age of garlic chives.

Tested Part of the Plant	Chlorophyll a			Chlorophyll b			Total Chlorophyll		
	Season 2	Season 3	Mean	Season 2	Season 3	Mean	Season 2	Season 3	Mean
green stem	514.61 ± 2.72 ^bcd^*	455.54 ± 0.57 ^cd^	485.1 ^B^	139.35 ± 1.01 ^cde^	174.60 ± 0.31 ^bcd^	157.0 ^B^	714.36 ± 1.92 ^cd^	692.35 ± 1.96 ^cd^	703.4 ^B^
inflorescence	207.08 ± 0.15 ^ef^	302.74 ± 2.52 ^ef^	254.9 ^C^	85.05 ± 2.28 ^ef^	109.06 ± 1.31 ^def^	97.1 ^C^	325.30 ± 0.38 ^ef^	455.96 ± 2.93 ^ef^	390.6 ^C^
inflorescence stem	682.23 ± 2.64 ^ab^	632.44 ± 1.56 ^abc^	657.3 ^A^	237.06 ± 2.96 ^ab^	212.82 ± 1.56 ^abc^	224.9 ^A^	1018.54 ± 1.51 ^ab^	936.08 ± 0.40 ^ab^	977.3 ^A^
mean	468.0 ^A^	463.6 ^A^	-	153.8 ^A^	165.5 ^A^	-	686.1 ^A^	694.8 ^A^	-

* The results were expressed as the mean ± standard deviation (SD). Different lowercase letters indicate statistically significant differences (*p* ≤ 0.05) between the tested plant parts and plant ages, whereas different uppercase letters denote significant differences (*p* ≤ 0.05) for the main factor.

**Table 4:** Antioxidant capacity expressed as percentage inhibition of DPPH free radicals depending on the tested part and age of garlic chives.

Tested Part of the Plant	Season 2	Season 3	Mean
green stem	45.83 ± 0.28 ^ef^*	43.39 ± 0.12 ^ef^	44.6 ^C^
inflorescence	88.68 ± 1.29 ^ab^	90.85 ± 0.07 ^ab^	89.8 ^A^
inflorescence stem	74.16 ± 0.21 ^cd^	75.84 ± 0.08 ^cd^	75.0 ^B^
mean	69.6 ^A^	70.0 ^A^	-

* The results were expressed as the mean ± standard deviation (SD). Different lowercase letters indicate statistically significant differences (*p* ≤ 0.05) between the tested plant parts and plant ages, whereas different uppercase letters denote significant differences (*p* ≤ 0.05) for the main factor.

**Table 5:** The percentage composition of the essential oils obtained from different parts of garlic chives.

No.	Compounds	R_t_ [min]	RI	RI_Lit._	S	F	FB
1.	Furfural	4.59	829	830	0.25	0.34	0.50
2.	2-Ethylpyridine	4.87	840	836	- *	-	0.13
3.	*trans*-Hex-3-en-1-ol	5.08	849	851	-	0.16	-
4.	*cis*-Hex-3-en-1-ol	5.17	853	852	-	-	0.04
5.	Diallyl sulfide	5.21	855	854	-	-	0.05
6.	Methional	6.43	905	904	0.67	0.89	0.31
7.	Heptanal	6.54	908	907	-	-	0.21
8.	Allyl methyl disulphide	6.76	914	915	3.79	3.14	9.78
9.	Methyl propyl disulphide	7.20	927	926	0.41	0.14	1.23
10.	Methyl 1-propenyl disulphide	7.49	936	934	0.78	0.47	3.79
11.	Camphene	8.06	952	952	0.17	-	0.31
12.	Dimethyl trisulphide	8.57	967	969	6.50	4.72	12.84
13.	1-Octen-3-ol	9.03	980	979	-	-	0.27
14.	3-Octanol	9.53	995	995	-	-	0.10
15.	Decane	9.67	999	1000	0.47	-	-
16.	*p*-Cymene	10.60	1022	1025	-	-	0.09
17.	γ-Terpinene	12.22	1061	1062	0.17	0.22	0.09
18.	*trans*-2-Octen-1-ol	12.49	1067	1067	-	0.17	-
19.	*cis*-Linalool oxide	12.59	1070	1069	0.96	-	0.36
20.	Diallyl disulphide	12.91	1077	1077	7.33	6.86	10.00
21.	*trans*-Linalool oxide	13.25	1086	1085	0.96	-	0.41
22.	Allyl propyl disulphide	13.47	1091	1088	2.11	3.52	1.93
23.	Linalool	13.79	1099	1098	8.00	3.16	8.25
24.	Nonanal	13.96	1102	1102	0.23	0.58	0.16
25.	Dipropyl disulphide	14.05	1105	1105	0.88	0.74	0.65
26.	*trans*, *trans*-2,4-Octadienal	14.41	1113	1113	-	-	0.17
27.	*cis*-*p*-Menth-2-en-1-ol	14.83	1122	1123	-	0.15	0.28
28.	Allyl methyl trisulphide	15.48	1137	1135	15.26	11.58	17.28
29.	Camphor	15.67	1141	1143	0.74	-	0.30
30.	Methyl *trans*-1-propenyl trisulphide	15.83	1145	1144	0.08	-	-
31.	Methyl propyl trisulphide	15.90	1146	1148	0.48	0.18	0.40
32.	Methyl-1-(methylthio)ethyl disulphide	16.33	1156	1159	1.89	0.77	1.15
33.	Menthone	16.46	1159	1159	0.18	-	0.03
34.	*trans*-2-Nonenal	16.57	1162	1162	1.70	1.07	1.70
35.	1-Nonanol	16.90	1169	1169	0.16	-	0.19
36.	Terpinen-4ol	17.10	1174	1175	0.49	-	0.19
37.	Borneol	17.47	1182	1183	0.38	0.49	0.31
38.	α-Terpineol	17.69	1187	1187	0.96	0.28	0.41
39.	Methyl salicylate	17.85	1190	1190	0.42	0.26	0.34
40.	Estragole (Methyl chavicol)	18.06	1195	1196	1.96	-	0.87
41.	Myrtenol	18.41	1203	1203	0.20	0.34	-
42.	Decanal	18.56	1206	1206	0.38	-	0.37
43.	Dimethyl tetrasulphide	18.76	1211	1211	2.13	2.46	3.05
44.	*trans*-Carveol	19.37	1225	1224	0.29	-	0.12
45.	Pulegone	19.84	1236	1236	0.35	-	0.09
46.	Carvone	20.04	1240	1242	0.21	-	-
47.	Geraniol	20.55	1251	1250	1.14	0.53	0.51
48.	Piperitone	20.92	1260	1262	0.27	0.32	0.14
49.	2-Phenyl-2-butenal	21.25	1268	1268	0.20	-	-
50.	Bornyl acetate	21.93	1283	1285	0.43	-	0.11
51.	*p*-Cymen-7-ol	22.12	1287	1287	-	0.19	0.03
52.	*cis*-Propenyl propyl trisulphide	22.19	1289	1289	1.18	-	0.57
53.	Diallyl trisulphide	22.52	1296	1298	4.32	5.71	4.28
54.	Di-2-propenyl trisulphide	22.58	1298	1297	0.58	-	0.17
55.	Tridecane	22.76	1302	1301	0.12	-	-
56.	Nonyl acetate	23.01	1308	1308	-	0.13	-
57.	3-Methoxyoctane	23.08	1309	1311	0.85	0.27	0.49
58.	4-Vinylguaiacol	23.42	1317	1315	0.25	0.34	0.19
59.	*trans*, *trans*-2,4-Decadienal	23.46	1318	1318	0.09	0.14	0.02
60.	Allyl propyl trisulphide	23.63	1322	1323	0.86	1.13	0.63
61.	Dipropyl trisulphide	23.97	1330	1328	0.83	0.51	0.30
62.	Bicycloelemene	24.18	1335	1336	-	0.16	-
63.	Di-1-propenyl trisulphide	24.74	1348	1347	0.06	-	-
64.	Eugenol	24.97	1354	1355	0.66	0.17	0.37
65.	Allyl methyl tetrasulphide	25.87	1375	1371	2.10	3.17	2.17
66.	Methyl cinnamate	26.02	1378	1379	0.26	-	-
67.	β-Cubebene	26.45	1388	1388	0.35	-	-
68.	β-Elemene	26.51	1390	1390	-	-	0.15
69.	β-Bourbonene	26.56	1391	1391	-	-	0.07
70.	*cis*-Jasmone	26.70	1394	1394	0.06	0.18	0.20
71.	Longifolene	27.28	1408	1407	0.40	0.47	0.30
72.	β-Caryophyllene	27.56	1415	1415	0.34	-	0.02
73.	*trans*-α-Ionone	27.94	1425	1426	-	0.15	0.18
74.	β-Gurjunene	28.24	1432	1434	0.05	-	-
75.	*trans*-α-Bergamotene	28.46	1437	1438	-	2.15	0.05
76.	*trans*-Geranylacetone	28.96	1450	1451	0.40	0.28	0.59
77.	α-Elemene	29.63	1466	1469	0.31	0.24	0.49
78.	*trans*-β-Ionone	30.28	1482	1482	0.39	-	0.17
79.	Germacrene D	30.53	1488	1487	0.16	-	0.03
80.	2-Tridecanone	30.68	1492	1494	-	0.13	-
81.	α-Selinene	30.89	1497	1498	0.23	-	0.02
82.	Bicyclogermacrene	31.08	1502	1505	0.79	0.89	0.37
83.	α-Farnesene	31.34	1509	1509	0.30	0.22	0.11
84.	Diallyl terasulphide	32.31	1533	1538	0.62	0.93	0.49
85.	Di-2-propenyl tetrasulphide	32.59	1541	1541	-	0.19	-
86.	*trans*-Nerolidol	33.61	1567	1568	-	-	0.09
87.	Dipropyl tetrasulphide	33.78	1572	1573	0.07	0.20	0.17
88.	*cis*-3-Hexenyl benzoate	34.02	1578	1576	0.09	-	-
89.	Caryophyllene oxide	34.53	1591	1592	0.12	0.21	0.13
90.	Guaiol	34.80	1598	1597	-	0.19	0.89
91.	β-Oplopenone	35.16	1608	1608	-	0.17	-
92.	Humulene epoxide II	35.30	1611	1609	-	0.18	0.17
93.	1,10-di-epi-Cubenol	35.56	1618	1619	0.45	0.27	0.79
94.	epi-α-Cadinol	36.21	1636	1638	-	-	0.12
95.	α-Muurolol	36.42	1642	1642	-	2.61	-
96.	τ-Muurolol	36.74	1651	1651	0.05	-	-
97.	α-Eudesmol	37.05	1659	1656	0.06	-	-
98.	1-Tetradecanol	37.52	1672	1672	0.49	1.07	0.39
99.	epi-α-Bisabolol	38.28	1692	1692	-	-	0.05
100.	Pentadecanal	38.73	1705	1707	0.09	-	-
101.	5-Ethyl-5-Methylpentadecane	38.88	1709	1710	-	0.21	-
102.	α-Sinensal	40.46	1754	1752	-	0.15	0.65
103.	Benzyl benzoate	40.58	1758	1759	-	0.43	-
104.	2-Methylheptadecane	40.77	1763	1764	-	-	0.07
105.	Myristic acid	40.87	1766	1769	0.19	0.31	0.23
106.	*trans*-α-Atlantone	41.00	1770	1773	-	-	0.13
107.	3-Methylheptadecane	41.12	1773	1774	-	-	0.05
108.	Ethyl myristate	41.90	1795	1795	0.12	-	0.05
109.	Hexadecanal	42.43	1811	1811	-	0.12	-
110.	Isopropyl myristate	43.15	1832	1831	0.48	1.09	0.22
111.	Hexahydrofarnesyl acetone	43.62	1846	1845	-	0.28	0.10
112.	4-Methylpentadecane	44.18	1863	1860	0.65	-	0.48
113.	Pentadecanoic acid	44.54	1874	1873	0.22	-	-
114.	1-Nonadecene	45.23	1890	1894	0.16	-	-
115.	Methyl palmitate	46.09	1921	1922	0.18	-	-
116.	Dibutyl phthalate	47.22	1957	1957	-	0.66	0.32
117.	Palmitic acid	47.36	1961	1962	4.32	-	-
118.	1-Heptadecanol	48.18	1986	1986	0.41	0.94	0.12
119.	7,7-Diethylheptadecane	48.27	1989	1988	0.39	-	0.13
120.	Sulfur (S8)	48.74	2004	2004	0.19	0.32	0.19
121.	13-epi-Manool	49.03	2014	2013	-	0.12	-
122.	Isopropyl palmitate	49.24	2021	2023	0.56	0.27	0.17
123.	Heptadecanoic acid	50.95	2076	2077	0.26	0.15	0.25
124.	Methyl linoleate	51.29	2088	2090	0.30	-	-
125.	Methyl linolenate	51.48	2094	2098	0.81	0.53	0.09
126.	Phytol	51.84	2106	2109	0.67	0.48	0.11
127.	Nonadecanal	52.12	2115	2110	0.16	0.13	0.02
128.	Methyl stearate	52.42	2126	2128	0.35	-	-
129.	Linoleic acid	52.59	2131	2130	0.57	-	-
130.	Linolenic acid	52.79	2138	2143	0.27	0.95	0.04
131.	Ethyl linoleate	53.30	2155	2155	0.84	-	0.07
132.	Ethyl linolenate	53.49	2162	2166	1.63	-	0.11
133.	1-Nonadecanol	53.70	2169	2172	0.18	-	0.23
134.	1-Docosene	54.38	2192	2190	-	0.35	-
135.	Eicosanal	55.07	2216	2219	-	0.23	-
136.	*trans*-5-Eicosene	56.97	2284	2286	-	0.92	-
137.	1-Tricosene	57.20	2292	2294	0.23	5.82	0.31
138.	1-Tetracosene	59.88	2391	2394	-	1.14	-
139.	5,5-Dimethylheneicosane	60.63	2418	2410	-	0.28	-
140.	Docosanal	60.88	2429	2430	-	0.80	-
141.	11-Tricosene	62.22	2480	2480	-	0.23	-
142.	1-Pentacosene	62.50	2491	2492	0.73	7.51	0.24
143.	3-Methylpentacosane	64.56	2573	2573	-	0.63	-
144.	1-Hexacosene	64.99	2590	2593	0.05	0.60	-
145.	6-Methylhexacosane	66.35	2646	2647	0.06	-	-
146.	2-Methylhexacosane	66.79	2664	2662	-	0.26	-
147.	3-Methylhexacosane	67.23	2683	2686	-	0.26	-
148.	1-Heptacosene	67.41	2690	2694	1.33	5.22	0.14
149.	Methyl lignocerate	67.99	2715	2714	-	0.42	-
150.	5,17-Dimethylheptacosane	69.72	2788	2786	-	0.34	-
151.	Squalene	70.41	2819	2819	0.22	-	-
152.	2-Methyloctacosane	71.22	2854	2860	-	0.28	-
153.	1-Nonacosene	72.00	2889	2885	0.61	2.06	-
154.	1-Triacontene	74.22	2990	2998	-	0.30	-
	No. of identified compounds				102	91	102
	Grouped compounds [%]						
	Monoterpene hydrocarbons				0.34	0.22	0.49
	Oxygenated monoterpenes				15.96	5.74	12.13
	Phenols				0.91	0.51	0.56
	Phenylpropanoids				1.96	-	0.87
	Sesquiterpene hydrocarbons				2.93	4.13	1.61
	Oxygenated sesquiterpenes				0.68	4.06	3.12
	Diterpenoids				0.67	0.60	0.11
	Triterpenoids				0.22	-	-
	Sulfur compounds				53.12	45.28	71.43
	Fatty acids				5.83	1.41	0.52
	Fatty acids esters				5.27	2.31	0.71
	Aliphatic hydrocarbons				4.80	26.41	1.42
	Aldehydes				3.10	3.41	3.15
	Alcohols				1.24	2.34	1.34
	Esters				0.77	1.48	0.66
	Others				1.30	0.73	1.17
	Total identified [%]				99.10	98.63	99.29

RI: Retention indices relative to n-alkanes (C_7_–C_30_) on HP-5MS capillary column; RI_Lit._: retention index taken from NIST (https://webbook.nist.gov/chemistry/, accessed on 15 November 2025) or literature [43,44]. *: not detected; S—green stem; F—inflorescence; FB—stem with flower buds.

**Table 6:** The content of sulfur compounds in the essential oils obtained from different parts of garlic chives.

Essential Oil Constituent	Parts of the Plant		
	S	F	FB
Diallyl sulphide	0.00 *	0.00	0.05
Methional	0.67 b	0.89 a	0.31 c
Allyl methyl disulphide	3.79 b	3.14 b	9.78 a
Methyl propyl disulphide	0.41 b	0.14 c	1.23 a
Methyl 1-propenyl disulphide	0.78 b	0.47 c	3.79 a
Dimethyl trisulphide	6.50 b	4.72 c	12.84 a
Diallyl disulphide	7.33 b	6.86 b	10.00 a
Allyl propyl disulphide	2.11 a	1.17 b	1.93 a
Dipropyl disulphide	0.88 a	0.74 b	0.65 b
Allyl methyl trisulphide	15.26 a	11.58 b	17.28 a
Methyl *trans*-1-propenyl trisulphide	0.08	0.00	0.00
Methyl propyl trisulphide	0.48 a	0.18 b	0.40 ab
Methyl-1-(methylthio)ethyl-disulphide	1.89 a	0.77 c	1.15 b
Dimethyl tetrasulphide	2.13 b	2.46 b	3.05 a
*cis*-Propenyl propyl trisulphide	1.18 a	0.00 c	0.57 b
Diallyl trisulphide	4.32 b	5.71 a	4.28 b
Di-2-propenyl trisulphide	0.58 a	0.00 c	0.19 b
Allyl propyl trisulphide	0.86 ab	1.13 a	0.63 b
Dipropyl trisulphide	0.83 a	0.51 b	0.30 c
Di-1-propenyl trisulphide	0.06	0.00	0.00
Allyl methyl tetrasulphide	2.10 b	3.17 a	2.17 b
Diallyl terasulphide	0.62 b	0.93 a	0.49 c
Di-2-propenyl tetrasulphide	0.00	0.19	0.00
Dipropyl tetrasulphide	0.07 n.s.	0.20 n.s.	0.17 n.s.
Sulfur (S8)	0.19 b	0.32 a	0.19 b
Total [%]	53.12	45.28	71.43

Means followed by the same letter are not significantly different at *p* ≤ 0.05. 0.00 *—compound not detected in the essential oil. S—green stem; F—inflorescences; FB—stem with flower buds.

**Table 7:** Meteorological data covering the years of research 2023–2024 (data source: https://www.ogimet.com/, accessed on 20 October 2025).

Months	Mean Daily Temperature (°C)		Total Rainfall (mm)		Insolation (h)		Multiyear 1991–2020		
	2023	2024	2023	2024	2023	2024	Mean Daily Temperature (°C)	Total Rainfall (mm)	Insolation (h)
I	4.0	1.6	56	46	28	49	0.6	50.0	42.7
II	2.6	- *	50	-	91	-	1.5	32.8	66.7
III	5.2	7.9	52	20	118	140	4.2	38.4	121.2
IV	8.0	10.8	24	38	178	186	9.2	31.2	199.3
V	13.2	16.8	7	59	324	295	13.6	55.8	244.5
VI	18.1	17.7	63	70	281	249	16.8	60.3	242.3
VII	18.7	19.4	69	54	251	268	18.9	76.2	246.3
VIII	19.2	19.8	76	34	248	285	18.5	60.3	230.3
IX	15.3	16.7	58	33	211	233	14.3	47.7	160.0
X	10.1	11.0	43	26	176	129	9.5	43.5	105.7
XI	5.8	5.4	67	37	131	35	4.9	39.0	47.4
XII	2.1	4.3	52	33	97	20	1.9	43.0	32.2

* Explanations: no data available.

## Data Availability

The data presented in this study are available upon request from the corresponding author.
